# 883. The Current State of Access: Inequitable Access to Infectious Disease Physicians in the Time of the COVID-19 Pandemic and Declining Fellowship Match Rates

**DOI:** 10.1093/ofid/ofad500.928

**Published:** 2023-11-27

**Authors:** Benjamin P Fuller, Andrew Strumpf, Kathleen A McManus

**Affiliations:** University of Virginia Health, Charlottesville, Virginia; University of Virginia, Charlottesville, Virginia; University of Virginia, Charlottesville, Virginia

## Abstract

**Background:**

Equitable access to specialty care is a key component of population health. Guidelines recommend specialty care within a 60-minute (min) drive. In 2017, 80% of United States (US) counties had no Infectious Diseases (ID) physicians. In the setting of the COVID-19 pandemic and declining fellowship match rates, we characterized inequities in geographic access to ID physicians in the US.

**Methods:**

We geocoded the location of ID physicians from 2020 Medicare Provider Utilization and Payment Data. For people in the contiguous US, we conducted a cross-sectional analysis of one-way drive times to the closest ID physician overall and by age, gender, racial, and ethnic subgroups using 2020 US Census data. We calculated the median drive times and the proportion of each subgroup within x minutes to the nearest ID physician.

**Results:**

We identified 6000 ID physicians at 2582 locations (Fig 1). Fig 2 demonstrates one-way drive times. In the US, 7.9% of people live > 60 min from ID specialty care. When stratified by rural/urban, 46.7% of rural people and 1.6% of urban people live > 60 min from the closest ID physician (Fig 3). Rural people had a median drive time of 57.2 min (95% CI 56.6-57.9) while urban people had a median drive time of 10.9 min (10.8-11.0). For people who identify as American Indian and Alaska Native (AIAN), 70.3% in rural areas and 8.8% in urban areas live > 60 min from the closest ID physician. For rural AIAN populations, median drive time was 95.1 min (86.0-104.1). For rural populations, the median drive time was > 60 min for those who identify as Native Hawaiian and Other Pacific Islander (69.1; 64.5-75.8), multiple races (65.5; 63.7-67.6), or Hispanic (66.9; 65.0-68.7).

**Figure:**
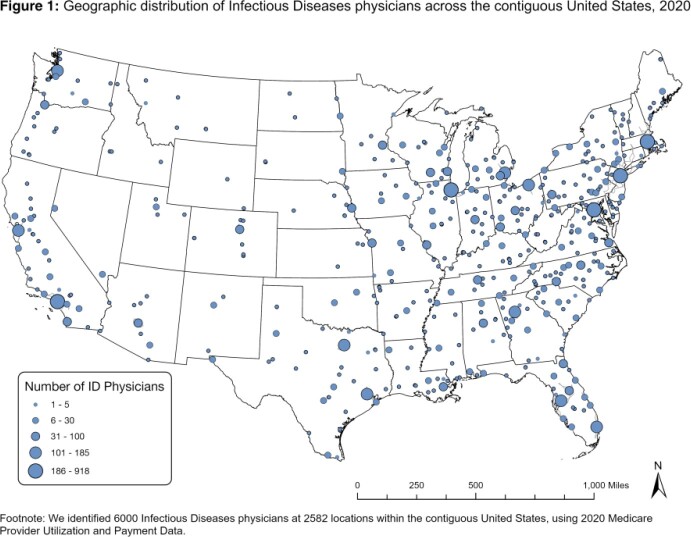

**Figure:**
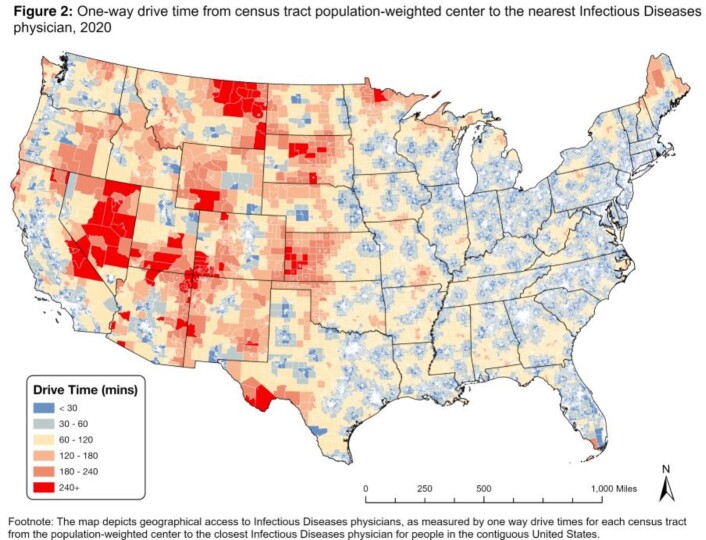

**Figure:**
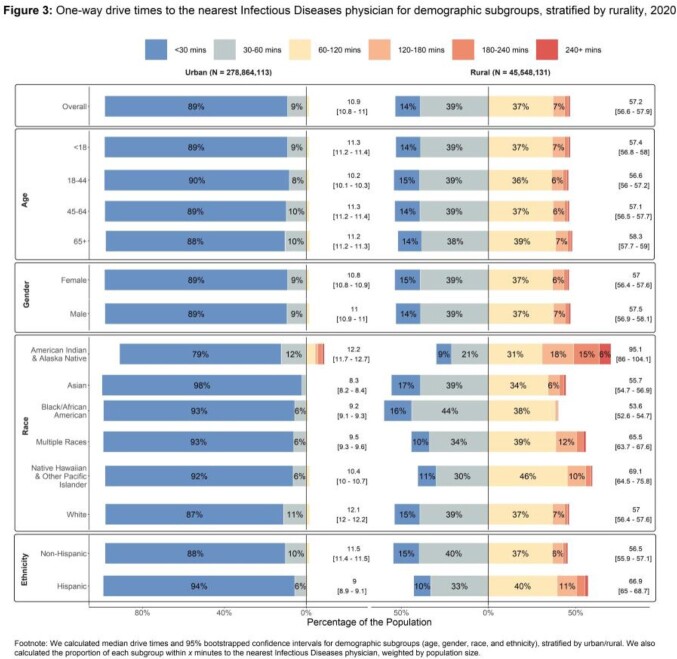

**Conclusion:**

Access to ID specialty care is inequitable. The largest disparities exist between rural and urban populations. Significant disparities exist for the AIAN population. In ID, we need recruitment and retention of our current workforce and recruitment of people who will serve rural and AIAN communities. If telemedicine continues to be supported, it could help to reduce disparities. This is also dependent on equitable access to high-speed internet, which is concentrated in urban/suburban areas. Without initiatives to expand the ID workforce and to increase internet access in rural areas, disparities in access will not improve.

**Disclosures:**

**Kathleen A. McManus, MD, MSCR**, Gilead Sciences, Inc.: Stocks/Bonds

